# RETRACTION: The Efficacy and Safety of Non‐Surgical Treatment of Diabetic Foot Wound Infections and Ulcers: A Systemic Review and Meta‐Analysis

**DOI:** 10.1111/iwj.70615

**Published:** 2025-04-21

## Abstract

**RETRACTION:** CaiX. , FangX. , WuL. , and MengX. , “The Efficacy and Safety of Non‐Surgical Treatment of Diabetic Foot Wound Infections and Ulcers: A Systemic Review and Meta‐Analysis,” International Wound Journal 21, no. 2 (2024): e14615, 10.1111/iwj.14615.38379242 PMC10827650

The above article, published online on 30 January 2024, in Wiley Online Library (http://onlinelibrary.wiley.com/), has been retracted by agreement between the journal Editor in Chief, Professor Keith Harding; and John Wiley & Sons Ltd. Following an investigation by the publisher, all parties have concluded that this article was accepted solely on the basis of a compromised peer review process. The editors have therefore decided to retract the article. The authors did not respond to our notice regarding the retraction.

**RETRACTION:** X. Cai , X. Fang , L. Wu , and X. Meng , “The Efficacy and Safety of Non‐Surgical Treatment of Diabetic Foot Wound Infections and Ulcers: A Systemic Review and Meta‐Analysis,” International Wound Journal 21, no. 2 (2024): e14615, 10.1111/iwj.14615.38379242 PMC10827650

The above article, published online on 30 January 2024, in Wiley Online Library (http://onlinelibrary.wiley.com/), has been retracted by agreement between the journal Editor in Chief, Professor Keith Harding; and John Wiley & Sons Ltd. Following an investigation by the publisher, all parties have concluded that this article was accepted solely on the basis of a compromised peer review process. The editors have therefore decided to retract the article. The authors did not respond to our notice regarding the retraction.

